# Are *DSM‐5* and *ICD‐11* posttraumatic stress disorder measures assessing the same construct? Evidence from a Rasch‐based analysis

**DOI:** 10.1002/jts.70105

**Published:** 2026-07-12

**Authors:** Emre Han Alpay

**Affiliations:** ^1^ Department of Psychology Mersin University Mersin Türkiye; ^2^ Guidance and Psychological Counseling Practice and Research Center Mersin University Mersin Türkiye

## Abstract

This study examined whether *DSM‐5* and *ICD‐11* self‐report operationalizations of posttraumatic stress disorder (PTSD) are psychometrically equivalent. Using Rasch modeling, we compared the psychometric properties and convergent validity of the PTSD Checklist for *DSM‐5* (PCL‐5) and the International Trauma Questionnaire (ITQ) in a sample of 483 Syrian refugees residing in Türkiye (63.9% women, *M*_age_ = 31.60 years, *SD* = 11.48). Both instruments demonstrated acceptable model fit and reliability. However, the PCL‐5, *r* = .792, showed significantly stronger associations with depression than the ITQ, *r* = .691, *p* < .001, whereas associations with anxiety were comparable. Probable PTSD prevalence was higher using *DSM‐5* criteria (26.9%) than *ICD‐11* criteria (21.9%). A proxy score comprising PCL‐5 items mapped onto *ICD‐11* symptom clusters also correlated more strongly, *r* = .790, withdepression than the ITQ, *r* = .701, suggesting that differences in item content rather than item count drive the observed pattern. These findings indicate that the PCL‐5 and ITQ are not psychometrically equivalent. The PCL‐5 appears to capture a broader internalizing distress syndrome, whereas the ITQ provides a more parsimonious assessment of trauma‐specific symptoms. The results are specific to self‐report operationalizations and cannot be generalized to clinician‐administered diagnoses. The choice of assessment instrument may, therefore, meaningfully influence prevalence estimates, comorbidity patterns, and the interpretation of PTSD in trauma‐exposed populations.

Posttraumatic stress disorder (PTSD) is one of the most extensively studied psychological consequences of exposure to traumatic events, yet its conceptual boundaries remain the subject of ongoing debate. Despite substantial advances in understanding trauma‐related psychopathology, fundamental questions persist regarding whether PTSD represents a coherent, unitary construct or a heterogeneous syndrome shaped by diagnostic frameworks. This ambiguity is reflected in the coexistence of two major diagnostic systems, the *Diagnostic and Statistical Manual of Mental Disorders* (5th ed.; *DSM‐5*; American Psychiatric Association [APA], 2013) and the *International Statistical Classification of Diseases and Related Health Problems* (11th ed.; *ICD‐11*; World Health Organization [WHO], 2019), which define PTSD in substantially different ways.

The *DSM‐5* conceptualizes PTSD as a broad and multifaceted disorder comprising 20 symptoms organized across four clusters, including a range of symptoms reflecting negative alterations in cognitions and mood (APA, 2013). Although this expanded framework increases descriptive richness, it has also raised concerns regarding internal heterogeneity and conceptual overlap with other forms of psychopathology. This operationalization of PTSD has been criticized because it allows an extremely large number of symptom combinations to yield the same diagnosis, raising questions about whether the *DSM‐5* diagnostic framework reflects a coherent syndrome or a collection of loosely related symptoms (Galatzer‐Levy & Bryant, 2013; Miller & Resick, 2007). These concerns are further amplified by the inclusion of several nonspecific symptoms, such as sleep disturbance, concentration difficulties, irritability, negative beliefs, emotional numbing, and diminished interest, which overlap substantially with core features of depression and anxiety disorders (Afzali et al., 2017; Friedman et al., 2011). As a result, elevated rates of comorbidity, particularly with major depressive disorder, are frequently observed in *DSM*‐based PTSD definitions.

In contrast, the *ICD‐11* adopts a more parsimonious and theory‐driven approach that explicitly aims to minimize overlap with other psychiatric conditions and to capture the core features of trauma‐related responses. The *ICD‐11* PTSD diagnosis is defined by three symptom clusters, reexperiencing the traumatic event in the present, the avoidance of trauma reminders, and a persistent sense of current threat, with at least one symptom required from each cluster following exposure to an extreme stressor (Hyland et al., 2018; WHO, 2019). This streamlined structure substantially reduces the number of possible diagnostic presentations and reflects a deliberate effort to enhance diagnostic specificity and clinical utility (Galatzer‐Levy & Bryant, 2013; Shevlin et al., 2018). It remains an open empirical question, however, whether the narrower *ICD‐11* criteria achieve better discrimination between PTSD and comorbid conditions or whether they risk underidentifying individuals with genuine trauma‐related distress that manifests through a broader symptom profile.

Empirical evidence supports this distinction to a degree. Studies have consistently shown that *ICD‐11* PTSD criteria are associated with lower rates of comorbid depression and anxiety compared to *DSM*‐based definitions (Barbano et al., 2019; Shevlin et al., 2018; Wisco et al., 2016). For example, Shevlin et al. (2017) reported that 27.4% of individuals who met the *DSM‐5* PTSD criteria also met the criteria for depression and anxiety disorders, whereas this rate decreased to 21.0% when using *ICD‐11* criteria. Recent comparative research further suggests that *DSM‐5* PTSD symptoms tend to be more strongly associated with broader emotional distress, whereas *ICD‐11* symptoms demonstrate more specificity to core trauma‐related processes (Hansen et al., 2023).

A central question arising from these differences is whether self‐report measures of *DSM‐5* and *ICD‐11* PTSD symptoms yield equivalent assessments of trauma‐related psychopathology, or if differences in item content and diagnostic criteria result in meaningfully different measurement properties. Despite the importance of this question, much of the existing literature has relied on traditional psychometric approaches, such as confirmatory factor analysis (CFA) or total score comparisons. These approaches are mathematically related to *item response theory* (IRT) and provide complementary information but may obscure item‐level differences when comparing instruments with differing structures. As a specific IRT approach, Rasch modeling places both individuals and items on a common interval‐level metric, enabling sample‐independent estimation of item difficulty parameters and latent trait scores that are not influenced by differences in the number of items or response formats (Bond et al., 2021; Wright & Masters, 1982). These properties make Rasch modeling particularly well‐suited for the present comparison between the PTSD Checklist for *DSM‐5* (PCL‐5; Weathers et al., 2013) and the International Trauma Questionnaire (ITQ; Cloitre et al., 2018), which differ substantially in length and structure.

Forcibly displaced populations, such as Syrian refugees, represent a particularly relevant group for examining these measurement questions. Refugees are typically exposed to multiple cumulative traumatic stressors across displacement trajectories, including war zone exposure, persecution, the loss of home and social networks, and postmigration adversities (e.g., discrimination, economic hardship; Alpay et al., 2021; Kira et al., 2023). The resulting psychological profiles are often complex, with PTSD frequently co‐occurring with depression and anxiety (Blackmore et al., 2020; Fazel et al., 2005). This high comorbidity context makes it particularly important to examine whether *DSM‐5* and *ICD‐11* PTSD measures differ in their associations with general emotional distress. Furthermore, cultural factors may shape how trauma‐related symptoms are expressed and endorsed in refugee populations, underscoring the need to evaluate measurement properties in this specific context rather than relying solely on findings from Western clinical samples.

The present study addressed this gap by employing a Rasch modeling framework to compare the PCL‐5 and the ITQ within the same sample of Syrian refugees residing in Türkiye. Using the *partial credit model* (PCM; Masters, 1982), latent PTSD severity estimates were derived for both instruments and examined in relation to depressive and anxiety symptoms. Differences in the strength of these associations were formally tested using Steiger's method for comparing dependent correlations (Meng et al., 1992; Steiger, 1980). As a secondary analysis addressing the role of item content versus instrument differences, we constructed a PCL–ICD proxy score by extracting PCL‐5 items corresponding to *ICD‐11* symptom clusters, enabling a comparison of item content effects independent of instrument differences. Additionally, sex differences in PTSD symptom severity and differential item functioning across sex groups were examined to assess the cross‐group stability of the findings.

Based on prior theoretical and empirical work, it was hypothesized that (a) PCL‐5 and ITQ scores would be strongly but not perfectly correlated, reflecting substantial but incomplete overlap between their respective self‐report operationalizations of PTSD; (b) PCL‐5‐derived PTSD symptom severity would show stronger associations with depressive symptoms compared to ITQ‐derived estimates, consistent with the broader item content of the *DSM‐5* instrument; and (c) differences in associations with anxiety would be comparatively smaller, given that both measures include avoidance and hyperarousal components that overlap with anxiety‐related processes. By integrating Rasch modeling with external validity and secondary proxy analyses, the present study aimed to clarify whether differences between these two self‐report measures reflect alternative operationalizations of a common construct or meaningfully distinct measurement approaches to trauma‐related psychopathology.

## METHOD

### Participants and procedure

Participants were 483 adult Syrian refugees who had fled Syria during the civil war and were residing in Mersin, Türkiye, at the time of data collection. Mersin is a city with a high concentration of Syrian refugees, making it a suitable setting for accessing this population. The sample consisted of 308 women (63.9%) and 174 men (36.1%), with one missing value on the gender variable. Participants ranged in age from 18 to 69 years (*M* = 31.60, *SD* = 11.48). Regarding marital status, 214 participants (44.3%) were married, and 211 (43.7%) were single, whereas smaller proportions of the sample were widowed (*n* = 45, 9.3%) or divorced (*n* = 13, 2.7%). Data were collected between February 2022 and September 2023 in neighborhoods with a high density of Syrian residents. Participants were recruited using convenience and snowball sampling methods. Data collection was conducted through face‐to‐face interviews using paper‐and‐pencil questionnaires.

Undergraduate students enrolled at Mersin University, who were themselves Syrian, collected the data. This approach facilitated communication, increased trust, and improved participation rates within the target population. Inclusion criteria required participants to be at least 18 years of age and to have fled Syria following the onset of the Syrian civil war. Individuals were excluded if they had a psychotic disorder or neurological condition that would impair their ability to understand or respond to the survey questions. Prior to participation, all individuals were informed about the purpose of the study and provided informed consent. The study protocol was approved by the Ethics Committee of Mersin University. All procedures were conducted in accordance with the Declaration of Helsinki.

### Measures

#### *DSM‐5* PTSD symptoms

The PCL‐5 (Weathers, Litz, et al., 2013) is a 20‐item self‐report measure designed to assess the severity of PTSD symptoms in accordance with the *DSM‐5* criteria (APA, 2013). Items are grouped into four symptom clusters: intrusions (Criterion B; Items 1–5), avoidance (Criterion C; Items 6–7), negative alterations in cognitions and mood (Criterion D; Items 8–14), and alterations in arousal and reactivity (Criterion E; Items 15–20). Respondents rate the degree to which each symptom has bothered them over the past month on a 5‐point Likert scale ranging from 0 (*not at all*) to 4 (*extremely*). Total scores range from 0 to 80, with higher scores reflecting higher levels of symptom severity. Per official PCL‐5 scoring guidelines (Weathers, Litz, et al., 2013), a provisional PTSD diagnosis can be established in two ways: (a) a total score of 31 or above, which is the recommended primary method, as it tends to produce more reliable results, or (b) the *DSM‐5* diagnostic algorithm, which considers items with a rating of 2 or higher as being endorsed and requires at least one Criterion B symptom, one Criterion C symptom, two Criterion D symptoms, and two Criterion E symptoms. Both approaches are reported in the present study for transparency and to enable direct comparisons with the criterion‐specific ITQ scoring approach. In the present study, the Arabic version of the PCL‐5, validated by Ibrahim et al. (2018), was administered. Internal consistency in the current sample was excellent, Cronbach's α = .96.

#### *ICD‐11* PTSD symptoms

The ITQ (Cloitre et al., 2018) is a brief, self‐report instrument developed to assess PTSD and Complex PTSD according to *ICD‐11* criteria (WHO, 2019). The PTSD component consists of six items organized into three symptom clusters, with two items per cluster: reexperiencing in the present (Items 1–2), avoidance (Items 3–4), and sense of current threat (Items 5–6). Each item is rated on a 5‐point scale ranging from 0 (*not at all*) to 4 (*extremely*). A provisional *ICD‐11* PTSD diagnosis requires endorsement of at least one item from each of the three clusters with a rating of 2 or higher. Although the measure also includes items to assess functional impairment and complex PTSD (CPTSD) symptoms, the present study used only the six core PTSD items. Internal consistency in the current sample was good, Cronbach's α = .88.

#### Depressive symptoms

Depressive symptoms were assessed using the Arabic version (Sawaya et al., 2016) of the Patient Health Questionnaire–9 (PHQ‐9; Kroenke et al., 2001), a nine‐item self‐report measure. Respondents rate symptom frequency over the past 2 weeks on a 4‐point scale ranging from 0 to 3, with higher scores reflecting more severe depressive symptoms. A cutoff score of 11 was used to indicate probable depression, following Manea et al. (2012). In this study, the PHQ‐9 demonstrated good internal consistency, Cronbach's α = .88.

#### Anxiety symptoms

Anxiety symptoms were measured using the Arabic version (Sawaya et al., 2016) of the Generalized Anxiety Disorder Scale–7 scale (GAD‐7; Spitzer et al., 2006), a seven‐item self‐report scale. Each item is rated on a 4‐point scale ranging from 0 to 3, reflecting symptoms over the past 2 weeks. A cutoff score of 11 was used to indicate probable anxiety, following Plummer et al. (2016). In this study, the GAD‐7 demonstrated good internal consistency, Cronbach's α = .89.

### Data analysis

Before conducting the main analyses, descriptive statistics and Pearson correlations were computed for all study variables. Normality was evaluated using skewness and kurtosis values, with values within 2 (plus or minus) considered acceptable (George & Mallery, 2018). Missing data were minimal (i.e., less than 1%) and handled using listwise deletion.

The psychometric properties of the ITQ PTSD subscale and the PCL‐5 were examined using the PCM (Masters, 1982) implemented in the *TAM* package in R (R Core Team, 2023; Robitzsch et al., 2022). Item fit was evaluated using infit and outfit mean square (MNSQ) statistics, with values of 0.60–1.40 considered acceptable (Bond et al., 2021; Linacre, 2002). Person reliability was assessed using expected a posteriori (EAP) and weighted likelihood estimate (WLE) reliability coefficients.

Unidimensionality was evaluated using the standardized root mean square residual (SRMSR) and the mean absolute deviation of Q3 residuals (MADaQ3). Rasch‐calibrated theta scores were estimated using the WLE method, and Wright maps were generated to examine item–person targeting. To examine convergent validity, Pearson correlations were computed between PTSD theta scores (θ) and PHQ‐9 and GAD‐7 scores. Differences between dependent correlations were tested using Steiger tests (Meng et al., 1992; Steiger, 1980).

As a secondary analysis, a PCL–ICD proxy score was constructed using seven PCL‐5 items corresponding to *ICD‐11* PTSD symptom clusters. Correlations involving the proxy score were compared with those obtained for the full PCL‐5 and the ITQ. Gender differences in PTSD symptom severity were examined using independent samples *t* tests with Cohen's *d* effect sizes. Differential ıtem functioning (DIF) across gender was evaluated by comparing item difficulty estimates between women and men, with differences greater than 0.50 logits indicating potential DIF (Zwick, 2012).

## RESULTS

### Prevalence and comorbidity

Table 1 presents prevalence rates and comorbidity patterns across diagnostic approaches. Probable PTSD prevalence was higher according to the PCL‐5 (cutoff score: 26.9%, *DSM‐5* algorithm: 27.1%) than the ITQ (21.9%). High rates of comorbid depression and anxiety were observed for both frameworks, with the PCL‐5 showing slightly more overlap with depressive symptoms. All chi‐square tests were statistically significant, *p*s < .001.

**TABLE 1:** Prevalence rates and comorbidity patterns

**Variable**	***n***	**%**
PCL‐5 PTSD (total score ≥ 31)^a^	130	26.9
PCL‐5 PTSD (*DSM‐5* algorithm)^a^	131	27.1
ITQ PTSD (*ICD‐11* algorithm)^b^	106	21.9
Depression (PHQ‐9 ≥ 11)	155	32.1
Anxiety (GAD‐7 ≥ 11)	112	23.2
Depression + Anxiety	100	20.7
Depression + Anxiety + PCL‐5 PTSD (cutoff)^a^, ^c^	82	82.0
Depression + Anxiety + PCL‐5 PTSD (algorithm)^a^, ^c^	82	82.0
Depression + Anxiety + ITQ PTSD^b^, ^c^	68	68.0

*Note*. *N* = 483. PTSD = posttraumatic stress disorder; *DSM‐5* = *Diagnostic and Statistical Manual of Mental Disorders* (5th ed.); PCL‐5 = PTSD Checklist for *DSM‐5*; ITQ = International Trauma Questionnaire; *ICD‐11* = *International Statistical Classification of Diseases and Related Health Problems* (11th ed.); PHQ‐9 = Patient Health Questionnaire–9; GAD‐7 = Generalized Anxiety Disorder–7 scale.

^a^ Both PCL Checklist for *DSM‐5* (PCL‐5) scoring approaches are reported for transparency. Per official PCL‐5 guidelines (Weathers et al., 2013), the cutoff score method is recommended as the primary approach due to greater reliability; the *DSM‐5* algorithm is included as a robustness check. Results were virtually identical across methods (26.9% vs. 27.1%).

^b^ *ICD‐11* diagnostic algorithm for the ITQ requires a rating of 2 or higher on at least one item per symptom cluster.

^c^ Percentages reflect proportions within the Depression + Anxiety subgroup (*n* = 100).

### Rasch model fit: ITQ PTSD

The six‐item ITQ PTSD subscale was subjected to a PCM analysis, and the results indicated satisfactory model fit. Person reliability was acceptable, EAP = .858, WLE = .763, indicating that the scale can reliably differentiate between individuals with varying levels of PTSD symptom severity. The unidimensionality assumption was supported by an SRMSR value of .037 and a MADaQ3 value of .091, both of which were within acceptable thresholds. Item difficulty parameters (beta) ranged from 2.54 to 2.80 logits, reflecting a narrow difficulty range consistent with the scale's brevity and focus on core PTSD symptoms. All six items demonstrated acceptable infit and outfit MNSQ values within the recommended range of 0.60–1.40, suggesting adequate fit to the Rasch model. Item‐level statistics are presented in Table 2. The Wright map for the ITQ PTSD subscale is presented in Figure 1, illustrating the clustering of item difficulties at higher logit levels relative to the distribution of person theta scores.

**TABLE 2:** Comorbidity rates within probable posttraumatic stress disorder (PTSD) groups

	PCL‐5 (cutoff ≥ 31)^a^ *n* = 130	PCL‐5 (*DSM‐5* algorithm)^a^ *n* = 131	ITQ (*ICD‐11* algorithm) *n* = 106	PCL algorithm χ^2^(1, *N* = 483)	ITQ algorithm χ(1, *N* = 483)
Comorbidity	**%**	**%**	**%**		
Depression	85.4	83.2	81.1	212.30^***^	147.00^***^
Anxiety	66.2	68.7	70.8	205.57^***^	169.10^***^
Depression + Anxiety	63.1	62.6	64.2	188.66^***^	152.77^***^

*Note*: Percentages reflect proportions within each PTSD group. Chi‐square tests evaluated the association between PTSD diagnosis (positive vs. negative) and each comorbid condition across the full sample. PCL‐5 = PTSD Checklist for *DSM‐5*; *DSM‐5* = *Diagnostic and Statistical Manual of Mental Disorders* (5th ed.); ITQ = International Trauma Questionnaire; *ICD‐11* = *International Statistical Classification of Diseases and Related Health Problems* (11th ed.)

^a^ The prmary scoring method for the PCL‐5 is a cutoff score of 31 or higher, per official guidelines (Weathers et al., 2013). The PCL‐5 *DSM‐5* algorithm was included as a robustness check.

*** *p* < .001.

**FIGURE 1:**
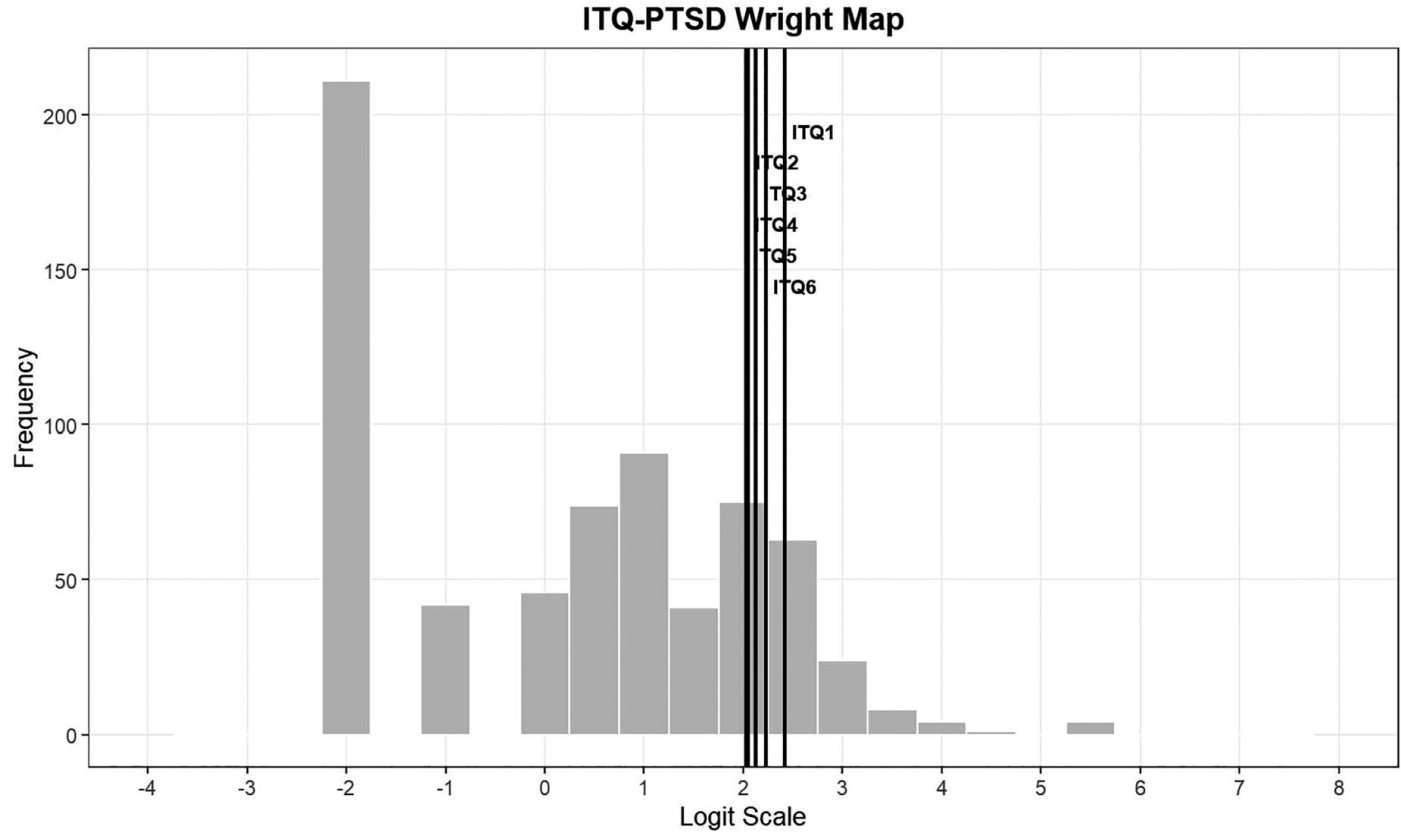
Wright map for the International Trauma Questionnaire (ITQ)–posttraumatic stress disorder (PTSD) scale (six items) *Note*: Vertical lines represent item difficulty parameters (unstandardized beta values) for each ITQ‐PTSD item. The histogram shows the distribution of person theta scores. All six items cluster between logit 2.54 and 2.8, above the majority of respondents, indicating that the items are relatively challenging for this sample.

### Rasch model fit: PCL‐5

The 20‐item PCL‐5 was similarly analyzed using a PCM analysis and demonstrated high person reliability, EAP = .918, WLE = .862, indicating strong measurement precision. Unidimensionality indices were within acceptable limits, SRMSR = .051, MADaQ3 = .110, though they were slightly higher compared to the ITQ, which is expected given the broader symptom coverage of the PCL‐5. Item difficulty parameters ranged from 1.75 to 2.49 logits, reflecting more variability compared to the ITQ. Most items (18 of 20) showed acceptable infit and outfit MNSQ values. However, two items demonstrated notable misfit: PCL19, outfit MNSQ = 1.40, and PCL20, outfit MNSQ = 1.13. Both items reflect hyperarousal (i.e., alterations in arousal and reactivity) symptoms, namely concentration difficulties and sleep disturbances, which are not specific to PTSD and frequently co‐occur with depressive and anxiety disorders in trauma‐exposed populations. This misfit is discussed further in relation to the convergent validity findings reported later. Item‐level statistics are reported in Table 3.

**TABLE 3:** Correlations between the PTSD Checklist for *DSM‐5* (PCL‐5), PCL‐5–*ICD* proxy, and the International Trauma Questionnaire (ITQ) and measures of depression and anxiety

Measure	Items	PHQ‐9 (*r*)	GAD‐7 (*r*)	ITQ (*r*)
PCL‐5 (full scale)	20	.847	.827	.787
PCL–*ICD* proxy^a^	7	.790	.782	.843
ITQ (*ICD‐11*)	6	.701	.757	—

*Note*: All correlations were statistically significant at *p* < .001. Steiger test comparing PCL–*ICD* proxy versus ITQ correlations with the Patient Health Questionnaire–9 (PHQ‐9): *t*(480) = 4.99, *p* < .001. PTSD = posttraumatic stress disorder; *DSM‐5* = *Diagnostic and Statistical Manual of Mental Disorders* (5th ed.); *ICD‐11* = *International Statistical Classification of Diseases and Related Health Problems* (11th ed.); GAD‐7 = Generalized Anxiety Disorder–7 scale.

^a^ Comprises seven PCL‐5 items corresponding to the three *ICD‐11* PTSD symptom clusters: reexperiencing (Items 1, 2, and 3), avoidance (Items 6 and 7), and sense of current threat (Items 17 and 18).

The Wright map for the PCL‐5 is presented in Figure 2, illustrating the broader distribution of item difficulties and the relative mismatch between item thresholds and person‐ability levels in this community‐based sample. Both Wright maps indicated that item difficulties were generally positioned above the mean person‐ability level, consistent with a community‐based sample in which most participants did not exceed clinical PTSD thresholds. The PCL‐5 showed a broader distribution of item difficulties than the ITQ.

**FIGURE 2:**
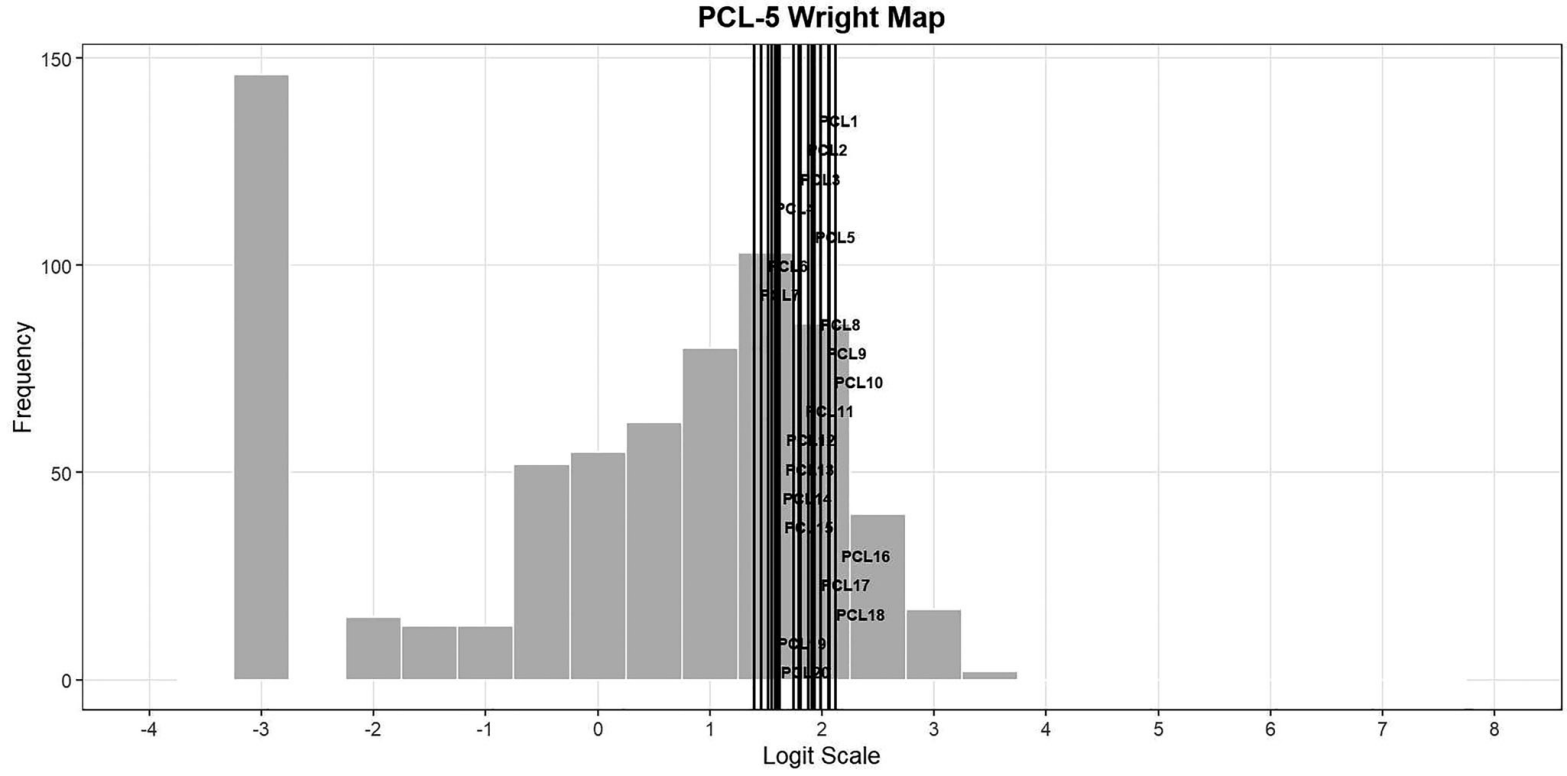
Wright map for the PTSD Checklist for DSM‐5 (PCL‐5) 20‐item scale *Note*: Vertical lines represent item difficulty parameters for each of the 20 PCL‐5 items. Items concentrate between logit 1.7 and 2.5, spanning a slightly wider range than the International Trauma Questionnaire (ITQ). The concentration of item difficulties in a narrow band, combined with the spread of person abilities toward lower logit values, indicates that many participants in this community‐based sample scored below the item difficulty thresholds. PTSD = posttraumatic stress disorder; *DSM‐5* = *Diagnostic and Statistical Manual of Mental Disorders* (5th ed.).

### Convergent validity: Correlations with depression and anxiety

To examine convergent validity, Rasch‐calibrated theta scores from both the ITQ and PCL‐5 were correlated with total PHQ‐9 and GAD‐7 scores. As shown in Table 4, both theta scores were substantially correlated with depression and anxiety measures, providing evidence of convergent validity for both scales. PCL‐5 theta scores showed a stronger association with PHQ‐9 depression scores, *r* = .792, compared to ITQ theta scores, *r* = .691. In contrast, the correlations with GAD‐7 anxiety scores were more similar across the two scales, PCL‐5: *r* = .760, ITQ: *r* = .740. The intercorrelation (*r*) between the two theta scores was .787, indicating strong but not perfect overlap.

**TABLE 4:** PTSD Checklist for *DSM‐5* (PCL‐5) item parameters and fit statistics

**Cluster and item**	**Description**	***B*** ^a^	**Infit**	**Outfit**
Intrusion				
PCL1	Intrusive memories	2.23	1.13	0.88
PCL2	Nightmares	2.15	1.14	1.01
PCL3	Flashbacks	2.05	1.00	0.83
PCL4	Emotional distress	1.91	1.05	0.80
PCL5	Physical reactivity	2.27	1.02	0.71
Avoidance				
PCL6	External avoidance	1.82	1.08	0.86
PCL7	Internal avoidance	1.75	1.02	0.78
NACM				
PCL8	Amnesia	2.36	1.06	0.69
PCL9	Negative beliefs	2.41	0.99	0.84
PCL10	Self‐blame	2.42	1.16	0.90
PCL11	Negative affect	2.21	0.87	0.69
PCL12	Anhedonia	2.01	0.99	0.92
PCL13	Detachment	2.01	1.00	0.85
PCL14	Emotional numbing	2.00	0.89	0.64
AAR				
PCL15	Irritability	1.99	0.90	0.78
PCL16	Risk behavior	2.49	0.97	0.64
PCL17	Hypervigilance	2.38	0.97	0.72
PCL18	Startle response	2.43	0.98	0.64
PCL19^b^	Concentration problems	1.99	1.24	1.40*
PCL20^b^	Sleep problems	1.96	1.21	1.13
EAP reliability = .918 WLE reliability = .862	SRMSR = .051	MADaQ3 = .110		

*Note*: Infit and outfit mean square (MNSQ) values between 0.60 and 1.40 indicate acceptable fit.

^a^ Item difficulty, in logits. PTSD = posttraumatic stress disorder; *DSM‐5* = *Diagnostic and Statistical Manual of Mental Disorders* (5th ed.); NACM = negative alterations in cognitions and mood; AAR = alterations in arousal and reactivity; SRMSR = standardized root mean square residual; EAP = expected a posteriori; WLE = weighted likelihood estimate; MADaQ3 = mean absolute deviation of Q3 residuals.

^b^ These items demonstrated borderline outfit misfit; both items assess hyperarousal symptoms (concentration difficulties and sleep disturbances) that are nonspecific to PTSD.

### Comparison of correlations: Steiger tests

To formally test whether the differences in correlations between the PTSD symptom measures (i.e., PCL‐5 and ITQ) and measures of depression and anxiety were statistically significant, paired Steiger tests were conducted; the results are summarized in Table 5. The PCL‐5 theta score demonstrated a significantly stronger correlation with depression (PHQ‐9) compared to the ITQ theta score, *t*(480) = 5.63, *p* < .001, Δ*r* = .101. However, the difference in correlations with anxiety (GAD‐7) was not statistically significant, *t* (480)= 1.06, *p* = .290, Δ*r* = .019. These results suggest that the PCL‐5 showed more overlap with depressive symptomatology compared to the more parsimonious *ICD‐11*–based ITQ.

**TABLE 5:** Intercorrelations among the International Trauma Questionnaire (ITQ) theta score, PTSD Checklist for *DSM‐5* (PCL‐5) theta score, Patient Health Questionnaire–9 (PHQ‐9) total score, and Generalized Anxiety Disorder–7 scale (GAD‐7) total score

Variable	1	2	3	4
1. ITQ (θ)	‐	.787*	.691*	.740*
2. PCL (θ)		‐	.792*	.760*
3. PHQ total			‐	.872*
4. GAD total				‐

*Note*: *N* = 483. All correlations are statistically significant at *p* < .001. PTSD = posttraumatic stress disorder; *DSM‐5* = *Diagnostic and Statistical Manual of Mental Disorders* (5th ed.)

### Secondary analysis: PCL–‐ICD proxy score

To examine whether observed differences reflect item content rather than item count, a PCL–ICD proxy score was computed using seven PCL‐5 items corresponding to *ICD‐11* PTSD symptom clusters. The *r* values for the correlations between the proxy and the PHQ‐9 and GAD‐7 were .790 and .782, respectively, falling between the correlations for the full PCL‐5 (PHQ: *r* = .847, GAD: *r* = .827) and the ITQ (PHQ: *r* = .701, GAD: *r* = .757). A Steiger test confirmed that, compared to the ITQ, the proxy was significantly more strongly correlated with depression, *t* (480) = 4.99, *p* < .001, despite drawing from the same instrument, suggesting that item content rather than item count drives the observed pattern. These results are presented in Table 6.

**TABLE 6:** Steiger tests comparing the PTSD Checklist for *DSM‐5* (PCL‐5) and the International Trauma Questionnaire (ITQ) theta score correlations with depression and anxiety

Criterion	Comparison 1	*r*	Comparison 2	*r*	Steiger test	*p*
Depression (PHQ‐9)	PCL–PHQ	.792	ITQ–PHQ	.691	*t*(480) = 5.63	< .001
Anxiety (GAD‐7)	PCL–GAD	.760	ITQ–GAD	.740	*t*(480) = 1.06	.290

*Note*: *N* = 483. Steiger tests evaluated whether correlations with a common criterion variable differed significantly between PCL‐5 theta and ITQ theta. PTSD = posttraumatic stress disorder; *DSM‐5* = *Diagnostic and Statistical Manual of Mental Disorders* (5th ed.); PHQ‐9 = Patient Health Questionnaire–9; GAD‐7 = Generalized Anxiety Disorder–7 scale.

### Gender comparisons

Independent samples *t* tests revealed no statistically significant differences between women and men on either ITQ theta scores, *t*(704) = ‐0.12, *p* = .901, *d* = ‐0.01, or PCL‐5 theta scores, *t*(704) = ‐0.66, *p* = .508, *d* = ‐0.06. Effect sizes were negligible for both scales. These results are presented in Table 7.

**TABLE 7:** Gender differences in International Trauma Questionnaire (ITQ) and PTSD Checklist for *DSM‐5* (PCL‐5) theta scores

Scale	Women		Men				
	*M*	*SD*	*M*	*SD*	*t*(480)	*p*	*d*
ITQ θ	0.43	2.20	0.45	2.11	−0.12	.901	−0.01
PCL‐5 θ	0.14	2.23	0.28	2.16	−0.66	.508	−0.06

*Note*: *N* = 482, women: *n* = 308, men: *n* = 174; there was one missing value for gender. Cohen's *d* values below 0.20 indicate negligible effect sizes. PTSD = posttraumatic stress disorder; *DSM‐5* = *Diagnostic and Statistical Manual of Mental Disorders* (5th ed.)

### DIF and gender‐stratified validity

DIF was evaluated by comparing item difficulty parameters estimated separately for women and men. For the ITQ, the largest between‐group difference in item difficulty was 0.44 logits (ITQ Item 1), with all remaining items showing differences below 0.25 logits. None of the ITQ items exceeded the conventional DIF threshold of 0.50 logits, suggesting that the ITQ functions equivalently across gender groups. For the PCL‐5, the majority of items showed gender differences below 0.40 logits. However, PCL10 showed a borderline difference of 0.53 logits, suggesting that this item may function slightly differently across gender groups. Given that this finding is based on a nonparametric comparison rather than a formal DIF test statistic, this finding should be interpreted cautiously. Gender‐stratified convergent validity analyses revealed a highly consistent pattern across groups, as shown in Table 8. In both women and men, the PCL‐5 theta score showed a higher correlation with depression compared to the ITQ theta score, whereas correlations with anxiety were comparable across the two scales.

**TABLE 8:** Gender‐stratified correlations between International Trauma Questionnaire (ITQ) and PTSD Checklist for *DSM‐5* (PCL‐5) theta scores with depression and anxiety

Comparison	Women (*n* = 308)	Men (*n* = 174)	Full sample	Steiger test	*p*
PCL θ–PHQ‐9	.783	.812	.792	*t*(480) = 5.63	< .001
ITQ θ–PHQ‐9	.676	.716	.691		
PCL θ–GAD‐7	.742	.798	.760	*t*(480) = 1.06	.290
ITQ θ–GAD‐7	.739	.741	.740		

*Note*. All reported correlations are statistically significant at p < .001. Steiger test results reflect the comparison between PCL‐5 theta and ITQ theta correlations with each criterion variable in the full sample. PTSD = posttraumatic stress disorder; *DSM‐5* = *Diagnostic and Statistical Manual of Mental Disorders* (5th ed.); PHQ‐9 = Patient Health Questionnaire–9; GAD‐7 = Generalized Anxiety Disorder–7 scale.

## DISCUSSION

The present study used a Rasch modeling framework to examine whether *DSM‐5* and *ICD‐11* PTSD self‐report measures yielded psychometrically equivalent assessments in a sample of Syrian refugees residing in Türkiye. The findings provide converging evidence that, although the PCL‐5 and ITQ are strongly related (*r* = .787), they are not fully interchangeable as self‐report operationalizations of PTSD. The data supported all three hypotheses. Importantly, these findings pertain to the measurement properties of two self‐report instruments and should not be interpreted as evidence of differences in the underlying PTSD constructs themselves, as no clinician‐administered reference standard was available.

Consistent with prior research, PTSD symptom scores derived from the PCL‐5 and ITQ were highly correlated, confirming substantial overlap between their respective operationalizations of PTSD (Hyland et al., 2017; Shevlin et al., 2018). The strong correlation indicates that the two instruments largely capture the same underlying dimension of trauma‐related distress. Nonetheless, this overlap was not perfect, and the observed differences in convergent validity patterns suggest that the PCL‐5 and ITQ are not fully interchangeable, even if they do not necessarily assess categorically distinct constructs. This distinction became particularly evident in the convergent validity analyses, where the PCL‐5 demonstrated a significantly stronger association with depressive symptoms compared to the ITQ, whereas both measures showed comparable associations with anxiety.

This pattern is consistent with the argument that the PCL‐5, by virtue of its broader item content, particularly Criterion D items reflecting negative alterations in cognition and mood, captures a wider range of internalizing distress that overlaps with depressive symptomatology (Friedman et al., 2011; Zoellner et al., 2014). The ITQ, by contrast, restricts its content to reexperiencing, avoidance, and sense of current threat, which are more closely tied to fear‐ and threat‐based trauma responses. Crucially, the secondary proxy analysis provides additional support for this interpretation: A PCL–ICD proxy score comprising only PCL‐5 items corresponding to *ICD‐11* clusters correlated more strongly with depression than the ITQ, despite using items drawn from the same instrument as the full PCL‐5. This pattern suggests that the difference is not simply an artifact of item count or instrument‐level factors but reflects genuine differences in item content between the two operationalizations. Notably, this pattern was observed at the level of Rasch‐calibrated interval‐level theta scores, which are not influenced by item count differences, providing a more precise test of the underlying measurement differences between the two instruments.

The Rasch analyses further reinforced these differences at the item level. The ITQ demonstrated a narrow range of item difficulties and strong unidimensionality indices, consistent with its focus on core PTSD symptoms and its theoretically motivated parsimonious structure. In contrast, the PCL‐5 showed greater variability in item difficulty and slightly weaker fit indices, reflecting its broader symptom coverage and the inherent heterogeneity introduced by including items from multiple conceptually distinct clusters. Notably, two PCL‐5 items (PCL19: concentration problems, PCL20: sleep disturbances) exhibited borderline outfit misfit. These items belong to the alterations in arousal and reactivity cluster but assess symptoms that are highly nonspecific and commonly associated with a wide range of emotional disorders, including depression and generalized anxiety (Harvey et al., 2011). Their misfit in the Rasch model suggests that they may capture general psychological distress rather than PTSD‐specific processes.

The Wright maps provided additional insight into the measurement properties of the two scales. Both instruments showed evidence of mistargeting, with item difficulties generally exceeding the average level of symptom severity in the sample. This pattern is expected in a community‐based refugee sample in which not all participants meet clinical PTSD thresholds—indeed, only 26.9% and 21.9% of participants exceeded the PCL‐5 and ITQ clinical cutoffs, respectively. However, the mistargeting was more pronounced for the ITQ, for which items clustered tightly at approximately 2.5–2.8 logits, reflecting their focus on prototypical, high‐severity PTSD presentations. This suggests that the ITQ may have limited sensitivity for detecting subclinical or mild PTSD symptomatology and may be better suited for clinical samples in which the base rate of diagnosable PTSD is higher. An alternative interpretation is that the lower depression overlap observed for the ITQ may partly reflect the measure's tendency to identify individuals with more prototypical or higher‐severity PTSD presentations due to its narrower and more restrictive symptom structure. From this perspective, differences between the ITQ and PCL‐5 may reflect not only variations in item content but also differences in the severity range captured by each instrument.

The prevalence findings further highlight meaningful differences between the two scoring approaches. The PCL‐5 yielded higher rates of probable PTSD (cutoff: 26.9%, *DSM‐5* algorithm: 27.1%) compared to the ITQ (21.9%), consistent with previous studies showing that the *ICD‐11* criteria produce lower prevalence estimates (Brewin et al., 2017; Cloitre et al., 2018). Notably, the near‐identical prevalence estimates across PCL‐5 scoring methods corroborate the official PCL‐5 guideline recommendation that the cutoff approach yields comparably reliable results. Regarding comorbidity, among individuals with probable PTSD by PCL‐5 criteria, 85.4% also had probable depression, compared to 81.1% among those who met the ITQ criteria. Although this difference is modest, it is consistent with the hypothesis that PCL‐5‐based identification captures a broader range of internalizing distress. It is important to note, however, that these findings rely solely on self‐report measures. As a result, it remains unclear whether the *DSM‐5* tends to identify too many individuals or whether the *ICD‐11* identifies too few. Both interpretations are possible. Future studies incorporating clinician‐administered interviews, such as the Clinician‐Administered PTSD Scale for *DSM‐5* (CAPS‐5; Weathers, Blake, et al., 2013), will be important for clarifying this issue.

Taken together, these findings have important theoretical and clinical implications. From a nosological perspective, the results support the view that the PCL‐5 and ITQ are not simply interchangeable self‐report operationalizations of PTSD, even though they largely converge on the same underlying dimension. The *DSM‐5*‐based PCL‐5 captures a broader internalizing distress syndrome, whereas the *ICD‐11*‐based ITQ targets a more narrowly defined, fear‐and‐threat‐based trauma response. This distinction matters not only theoretically but also practically: The choice of self‐report instrument may substantially influence prevalence estimates; comorbidity profiles; and, potentially, treatment selection. Clinicians and researchers working with refugee populations should be aware that PCL‐5–based assessments may identify a broader group with significant internalizing distress, whereas ITQ‐based assessments may more narrowly identify individuals with core trauma‐specific presentations. Neither approach is inherently superior; the optimal instrument depends on the clinical or research goals and whether sensitivity or specificity is prioritized.

The refugee context is also important when interpreting these findings. Syrian refugees living in Türkiye often continue to face multiple ongoing stressors, including discrimination, legal uncertainty, financial difficulties, and social exclusion. These chronic challenges may contribute not only to PTSD symptoms but also to broader psychological distress (Alpay et al., 2021; Kira et al., 2023). In this context, the higher depression–PTSD overlap observed with the PCL‐5 may partly reflect a genuine co‐occurrence of traumatic stress and chronic psychosocial adversity rather than solely a measurement artifact. Cultural factors may also shape symptom expression: In collectivistic societies, emotional suffering is often expressed through somatic and cognitive channels that align more closely with the *DSM‐5*’s broader symptom profile (Hinton & Lewis‐Fernández, 2011). Future research should examine whether the differential patterns observed here replicate across diverse displaced populations and cultural contexts.

Several limitations should be considered when interpreting these findings. First, the cross‐sectional design precludes causal inferences regarding the associations among PTSD, depression, and anxiety. Second, the use of self‐report measures may introduce shared method variance, which could inflate observed correlations. Critically, the overendorsement of symptoms on self‐report measures relative to clinician‐administered interviews is well‐documented, and the absence of a clinician‐administered gold standard (e.g., the CAPS‐5) means that it is not possible to determine which instrument more accurately reflects true PTSD prevalence in this sample. Third, the sample was recruited using convenience and snowball sampling methods in a single geographic location, which may limit generalizability. Fourth, although Rasch modeling provides valuable insights into measurement properties, the assumption of unidimensionality imposes a constraint that may oversimplify the inherently multidimensional nature of PTSD as defined by the *DSM‐5*. Fifth, the nonparametric approach used for DIF detection does not provide formal statistical tests of item bias, and the findings should, therefore, be treated as exploratory. Sixth, measures were administered in a fixed order without counterbalancing, which may have influenced response patterns; future studies should counterbalance measure order. Seventh, the PCL–ICD proxy score was constructed on conceptual grounds and does not perfectly operationalize the *ICD‐11* criteria; future studies should validate this mapping more formally. Finally, no evidence was available that the PCL‐5 cutoff score of 31 is optimally calibrated for this specific refugee population.

The present findings indicate that the PCL‐5 and ITQ, as self‐report instruments, are not psychometrically equivalent operationalizations of PTSD despite their strong convergence. The PCL‐5 captures a broader spectrum of posttraumatic distress that overlaps substantially with depressive symptomatology, a pattern that is partly attributable to the broader item content of the *DSM‐5* instrument and not merely to differences in item count. These results underscore the importance of carefully considering the choice of assessment instrument in both research and clinical practice, as this choice may meaningfully influence prevalence estimates, comorbidity patterns, and the interpretation of trauma‐related psychopathology. Future research should extend these findings using clinician‐administered interviews and longitudinal designs to clarify how *DSM‐5* and *ICD‐11* PTSD assessments differentially predict functional impairment, treatment response, and long‐term outcomes across diverse trauma‐exposed populations.

## AUTHOR CONTRIBUTIONS

**Emre Han Alpay**: conceptualization, ınvestigation, methodology, validation, writing—review and editing, formal analysis, data curation.

## AUTHOR NOTE

During the preparation of this manuscript, the author used AI‐assisted tools (Claude) to improve language clarity, organization, and readability. All content was critically reviewed and revised by the author, who takes full responsibility for the final version of the manuscript.

The author would like to thank all participants who generously contributed their time to this study.

## OPEN PRACTICES STATEMENT

This study was not preregistered. Deidentified data are publicly available on the Open Science Framework at https://doi.org/10.17605/OSF.IO/972FT. Requests for additional materials can be sent via email to the corresponding author.
